# 3D Bioprinting of osteochondral tissue substitutes – *in vitro*-chondrogenesis in multi-layered mineralized constructs

**DOI:** 10.1038/s41598-020-65050-9

**Published:** 2020-05-19

**Authors:** David Kilian, Tilman Ahlfeld, Ashwini Rahul Akkineni, Anne Bernhardt, Michael Gelinsky, Anja Lode

**Affiliations:** Centre for Translational Bone, Joint and Soft Tissue Research, Faculty of Medicine and University Hospital Carl Gustav Carus, TU Dresden, Dresden, Germany

**Keywords:** Preclinical research, Orthopaedics

## Abstract

For the generation of multi-layered full thickness osteochondral tissue substitutes with an individual geometry based on clinical imaging data, combined extrusion-based 3D printing (3D plotting) of a bioink laden with primary chondrocytes and a mineralized biomaterial phase was introduced. A pasty calcium phosphate cement (CPC) and a bioink based on alginate-methylcellulose (algMC) – both are biocompatible and allow 3D plotting with high shape fidelity – were applied in monophasic and combinatory design to recreate osteochondral tissue layers. The capability of cells reacting to chondrogenic biochemical stimuli inside the algMC-based 3D hydrogel matrix was assessed. Towards combined osteochondral constructs, the chondrogenic fate in the presence of CPC in co-fabricated and biphasic mineralized pattern was evaluated. Majority of expanded and algMC-encapsulated cells survived the plotting process and the cultivation period, and were able to undergo redifferentiation in the provided environment to produce their respective extracellular matrix (ECM) components (i.e. sulphated glycosaminoglycans, collagen type II), examined after 3 weeks. The presence of a mineralized zone as located in the physiological calcified cartilage region suspected to interfere with chondrogenesis, was found to support chondrogenic ECM production by altering the ionic concentrations of calcium and phosphorus in *in vitro* culture conditions.

## Introduction

Bioprinting – additive manufacturing with the inclusion of cells into the fabrication process – offers tremendous possibilities to the field of tissue engineering and therefore for personalized regenerative medicine. It provides the opportunity of combining different materials for hard and soft tissue types, various specific cell types and respective biologically active factors in a spatially defined pattern. This strategy enables researchers to design more specific tissue models and patient-individualized, tissue-adapted implants. This includes merging a hydrogel based on natural or synthetic polymers with cells prior to fabrication, resulting in a suspension defined as *bioink*^1^. Specific material features are required for extrusion-based printing of stable 3D structures. In general, a higher polymer content can lead to improved shape fidelity. However, the denser the resulting polymer network is, the less attractive this provided 3D environment is for encapsulated cells. Therefore, a balance is needed between shear-thinning, viscoelastic behavior for printability and a network density allowing both high shape fidelity and biocompatibility for embedded cells^2^. This can be achieved by applying different strategies for the generation of volumetric constructs which utilize internal (via material blending), external (via material combinations to hybrid scaffolds) or technological (through additional components of the technical printing environment) stabilization concepts^3,4^.

**Figure 1:**
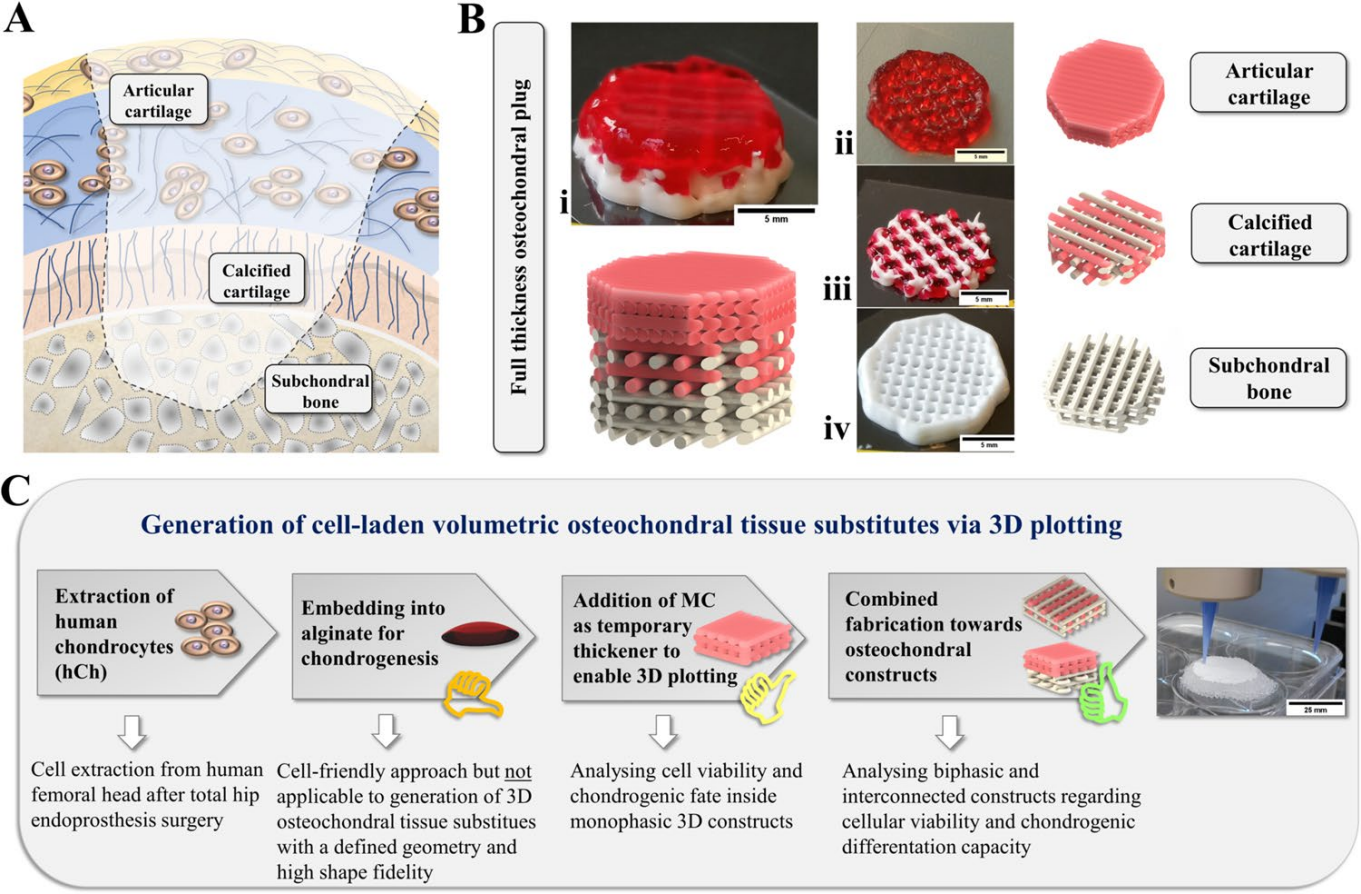
Tissue substitutes for multi-layered osteochondral defects via 3D plotting. (**A**) Multi-layered osteochondral tissue defects require zone-specific hierarchical repair strategies. (**B**) Multi-channel 3D plotting allows the fabrication of artificial full-thickness osteochondral plugs (i) based on a hydrogel (phenol red added for better visualization) and a calcium phosphate cement (white). A combination of cell-laden hydrogel and partly mineralized CPC-supported zones resembling articular cartilage (ii) and underlying layers, i.e. an interconnected network of calcified cartilage (iii) and a subchondral bone phase resembled by pure CPC (iv). Scale bar = 5 mm. (**C**) Scheme illustrating investigated experimental levels, regarding mono-/biphasic scaffold combinations and analysis paths throughout the study to achieve the generation of a volumetric hybrid scaffold based on interwoven or piled design, as well as individualized geometries (here: silicone model, scale bar = 25 mm).

One common orthopedic issue to be tackled by bioprinting strategies is an osteochondral defect affecting both bone- and cartilage-associated zonal compartments in human joints (Fig. 1A). Native tissue consists of different layers of chondrocytes embedded in an extracellular matrix (ECM). Cartilage ECM is typically formed by main component collagen type II of different fiber orientations and characterized by layers of different biochemical composition and cell density. This includes a calcified cartilage layer in close proximity to the underlying subchondral bone region^5^. Current treatment options of multi-layered defects as appearing in *osteochondritis dissecans* include the application of matrix-associated autologous chondrocyte transplantation (MACT)^6^, autologous iliac crest tissue or a combination of these clinical strategies^7,8^. However, no therapeutical option is available combining autologous patient specificity, patient-adjusted geometry and biofunctional properties of applied materials. Therefore, we aim for the application of patient-derived cells in a spatially defined manner (by 3D bioprinting), according to a design defined by internal and external architecture detected via magnetic resonance imaging (MRI), along with materials enabling matrix formation.

Tackling some of these aspects, hierarchical scaffolds of different designs including cell-laden and cell-free materials had been introduced earlier with the aim of recreating the zonal structure of articular cartilage^9,10^. Several studies suggested the application of biopolymer-based hydrogels for use in cartilage and osteochondral bioprinting: Gelatin-methacrylate (GelMA) hydrogels that have been described as one promising matrix for cartilage bioprinting^11^, were combined with microfibers to mimic the different zonal fiber alignments of articular cartilage^12^. Furthermore, different types of alginate-based scaffolds with^13^ and without cell encapsulation^14^ were suggested and successfully applied for cartilage regeneration *in vivo* and for tissue engineering studies before. Alginate hydrogels allow embedded human chondrocytes (hCh) or human mesenchymal stromal cells (hMSC) to undergo chondrogenesis in a 3D matrix^15–17^ without applying biofabrication approaches. Preventing an actual biological interaction to the embedded cells, it resembles a suitable environment for hCh which typically do not tend to adhere to provided matrix structures. However, as the low viscosity of alginate in cytocompatible concentrations does not allow extrusion-printing (3D plotting) with a good shape fidelity, Schütz *et al*. proposed the addition of methylcellulose as a thickener to temporarily increase the viscosity during printing. The blend consisting of 3% alginate and 9% methylcellulose (algMC) was suitable for precise plotting of volumetric constructs of clinically relevant dimensions and enabled encapsulation, survival and metabolic activity of hMSC at the same time^18^. Without the addition of methylcellulose, which is partially released from the crosslinked alginate network over time, the 3% alginate would not be 3D printable^18^. Other approaches presented a similar bioink composition for the use as cartilage or osteochondral tissue replacement due to its pore-forming properties after blending 2% alginate with methylcellulose of a viscosity of 400’000 cP in a ratio of 1:1 and 1:2^19^. Therefore, we chose for an algMC bioink as a valuable and potent option for the generation of osteochondral plugs – in combination with mineral phases.

Resembling the mineral zone of calcified cartilage^20^ inside the osteochondral interface (Fig. 1A), hydroxyapatite (HAp)-forming calcium phosphate cements (CPC) can play a promising role for use in osteochondral bioprinting approaches. Our group introduced the concept of multichannel plotting for co-extrusion of a mineralized biomaterial ink and bioprinting of cells: We combined the algMC-based bioink laden with hMSC with a clinically approved pasty CPC^21^. This material consists of a precursor powder with α-tricalcium phosphate (α-TCP) as main component and an oil-based carrier liquid to create an ink suitable for plotting without time limitation^22^. In contact with water, the setting reaction takes place that results in the formation of calcium-deficient nanocrystalline HAp^23,24^. HAp crystals feature osteoconductive characteristics for seeded cells *in vitro*^22,25^ or migrating cells that could remodel the degradable scaffold *in vivo*^26–28^. For this material, the generation of volumetric microporous structures with minimum feature sizes of approx. 200 µm and individually shaped geometries was proven^25,29,30^. The combination with plottable hydrogels, first described for high-concentrated alginate-based inks laden with bioactive factors^29,31^ and later for the algMC bioink^21^, is possible through alternating strand deposition which results in an interwoven network of both materials. For stabilization of such biphasic constructs, an adjusted two-step post-processing regime for CPC setting and alginate crosslinking had been established. This ensured viability of an embedded hMSC cell line and material microstability at the same time^21^. In the current study, these technical prerequisites are supposed to be applied to further develop this system to a fully biofunctional tissue substitues by evaluating its biological *in vitro* performance:

Our approach for bioprinting of osteochondral tissue substitutes involves a three-zone fabrication to build up a construct (Fig. 1Bi) from (1) a hCh-laden algMC part resembling the articular cartilage surface (ii), (2) a biphasic interwoven network zone for the calcified cartilage region based on the hCh-laden algMC and CPC (iii), and (3) a CPC-based subchondral bone part (iv) that can be potentially seeded with osteogenic (progenitor) cells after fabrication. Hence, to improve and replace concepts embedding patient-extracted cells in a non-printable alginate, we applied cells in a printable blend (algMC) that allows a spatially defined combination with the mineral phase (CPC) via multichannel plotting. With this, we created a path towards the generation of volumetric (thickness of 10 mm and more), open porous and biofunctional osteochondral tissue substitutes (Fig. 1C). Since bioprinting of algMC without supporting material enables the generation of stable volumetric structures, the CPC phase is not required as a supporting component for the cell-laden hydrogel; it mainly resembles the native bone matrix.

To study the suitability of algMC as artificial cartilage matrix, its impact on survival and fate of encapsulated hCh during redifferentiation was investigated towards the capability of cells forming chondrogenic ECM components. We aimed to describe the effect of the mineral phase on hCh redifferentiation by applying biphasic constructs in which the CPC and the cell-encapsulating algMC hydrogel were combined with close proximity and direct contact to each other. The CPC is calcium-deficient^23^, therefore binds calcium ions from the surrounding medium^32^ and changes the phosphate ion concentrations in its environment. In addition, the setting reaction includes an instable intermediate step of octacalcium phosphate which releases hydrogen ions causing an acidic shift of the surrounding chemical milieu^21,33,34^. Both aspects, ionic medium composition and pH milieu, might interfere with the capability of cells developing a chondrogenic phenotype which was evaluated in this study.

## Materials and Methods

### Chondrocyte extraction and expansion

**Figure 2:**
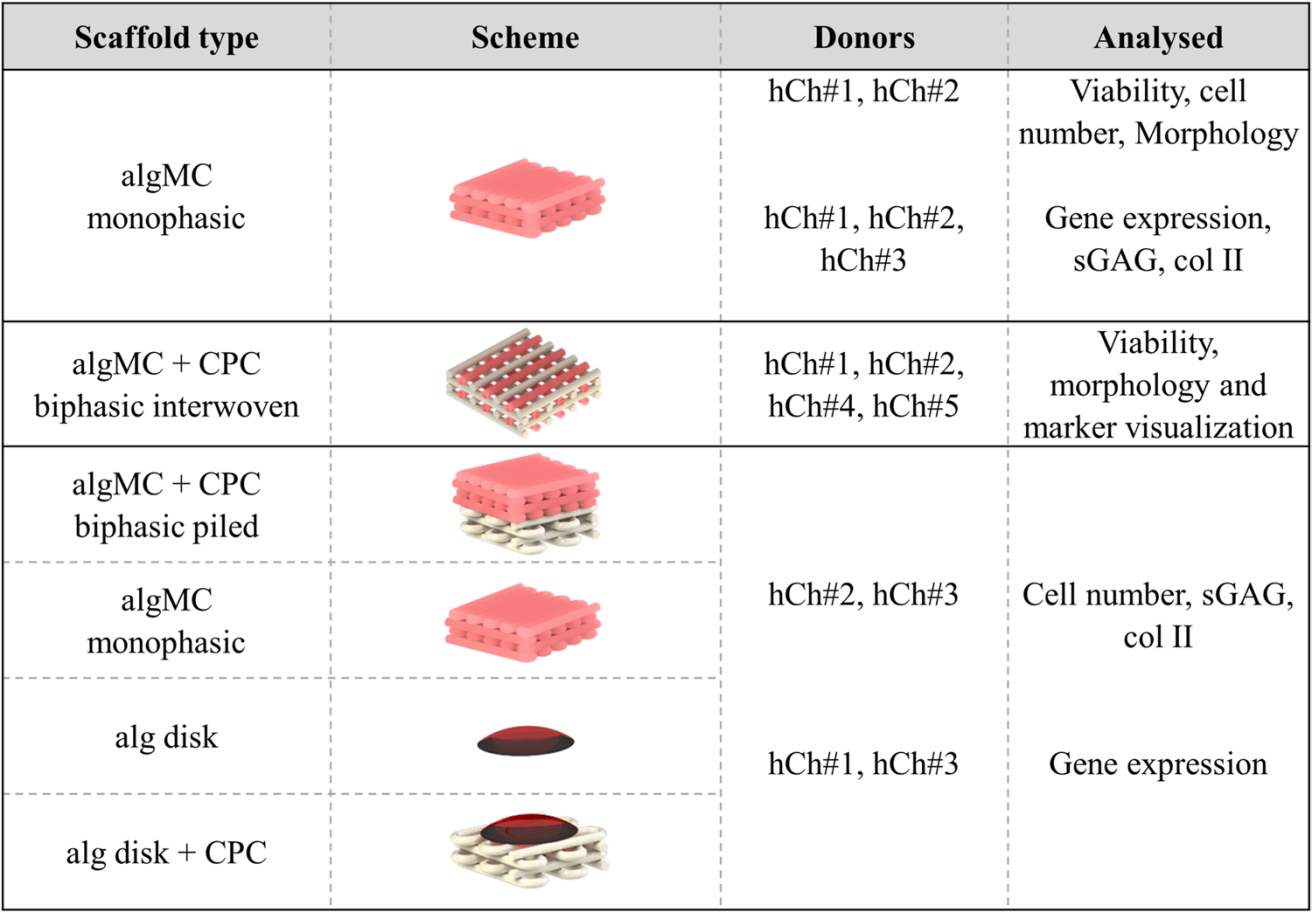
Summary of scaffold designs and experiments with different hCh donors (n = 5) in this study.

Human chondrocytes (hCh) were isolated from the full depth articular cartilage of femoral head of osteoarthritic patients undergoing total hip replacement at the University Hospital *Carl Gustav Carus* Dresden under aseptic conditions, by collagenase digestion for 15 h at 37 °C. Cells were harvested and expanded under standard cultivation conditions (5% CO_2_, 37 °C) up to passage 3 in Dulbecco’s Modified Eagle Medium (DMEM) with a D-glucose content of 1.0 g l^−1^, supplemented with 9% fetal calf serum (FCS) (Corning, USA) and 100 U ml^-1^ penicillin and 100 µg ml^-1^ streptomycin (P/S). For the bioprinting experiments, cells from five donors (3 females, 2 males, age 51–73) were used. For each analysis method, cells from at least two donors were used for replicating experiments (Fig. 2).

### Bioink preparation

An algMC blend, prepared as previously described^18,35^, was used for encapsulation of expanded primary hCh. In brief, 3 wt% alginic acid sodium salt from brown algae (Sigma-Aldrich, Germany) was dissolved in phosphate-buffered salt solution (PBS) (ThermoFisher, USA), autoclaved for sterilization (121 °C for 20 min in a Systec D-23 table-top autoclave), and 9 wt% of autoclaved methylcellulose powder (4,000 cP, Sigma Aldrich, USA) was added. After 2 h of methylcellulose swelling at RT, cells were added by gently blending them into the algMC ink in a ratio of 5 × 10^6^ suspended in 100 µl DMEM with 9% FCS, per 1 g of material.

### Biofabrication: 3D plotting and post-processing of monophasic and biphasic (interwoven and piled) scaffolds

A multi-channel extrusion printer BioScaffolder 3.1 (GeSiM mbH, Radeberg, Germany) equipped with two cartridges and mounted conical nozzle tips with an inner diameter of 410 µm (Nordson EFD GmbH, Germany) was used for fabrication of artificial tissue structures. The dimensions of square-shaped scaffolds consisting of 4 layers (total height ca. 1.3 mm) were chosen in the range of 7–8 mm, with a mean strand spacing of 600–1000 µm resulting in macroporous monophasic and biphasic scaffolds. CPC precursor powder and carrier liquid were provided by INNOTERE GmbH, Germany. An overview of applied scaffold designs, chondrocyte donors and experimental set-ups is given in Fig. 2.

#### Monophasic scaffolds

Monophasic scaffolds were fabricated by strandwise deposition of the hCh-laden algMC bioink with a dosing pressure of 90 kPa and a printing speed of 10 mm s^-1^. Immediately after plotting, they were stabilized by alginate crosslinking in 100 mM CaCl_2_ for 10 min.

#### Biphasic interwoven scaffolds

For biphasic interwoven scaffolds towards examination of impact of direct cell- and hydrogel-to-CPC contact, both materials were deposited in alternating fashion (Fig. 1B iii) (printing parameters for CPC: 100 kPa, 11 mm s^-1^); two-step post processing to enable crosslinking and setting reactions was applied as previously described^21^. This procedure includes incubation for 30 min at 37 °C in water-saturated atmosphere, followed by 10 min of ionic crosslinking in 100 mM CaCl_2_ before adding cell culture medium; the initial pre-setting reaction of the CPC phase in water-saturated atmosphere reduced the risk of microcrack formation in the cement strands^21^.

#### Biphasic piled scaffolds

To investigate the effect of the CPC zone on 3D chondrogenesis, 3D plotted hCh-laden algMC scaffolds after 30 min incubation at 37 °C in humid atmosphere and 10 min of CaCl_2_ crosslinking, were placed on top of simultaneously prepared CPC scaffolds of identical architecture and processing. This piling setup enabled separation of the phases for biochemical assays and gene expression analysis after 21 days. Both scaffolds were 3D plotted with a square base geometry (7.0 × 7.0 mm), a height of 4 layers and strand spacing of 700 µm, so that initial cell number in one algMC scaffold was 6.5–7.0 × 10^5^ cells. As standard reference model for chondrogenesis, a 1.2% alginate gel (alg) in PBS of equal cell density, with and without contact to a CPC scaffold, was included in the study to evaluate the impact of methylcellulose supplementation and a stable 3D network. Alg disks were prepared by pipetting 50 µl into the well prior to crosslinking in 100 mM CaCl_2_ for 10 min after 30 min incubation at 37 °C in humid atmosphere.

### Cultivation of bioprinted monophasic and biphasic (interwoven and piled) constructs

After 2 h of re-equilibration to culture conditions in DMEM, 1 ml of respective culture medium was added: To drive the expanded cells from their dedifferentiated progenitor state towards redifferentiation, samples were cultivated in chondrogenic differentiation (diff) medium consisting of DMEM with a D-glucose content of 4.5 g l^−1^ supplemented with P/S, 120 µM ascorbic acid 2-phosphate (AAP) (Sigma-Aldrich, USA), 40 µg ml^−1^ l-proline (Sigma-Aldrich, USA), 10^−7^ M dexamethasone (Sigma-Aldrich, USA), ITS + 1 (Insulin-transferrin-sodium selenite, linoleic-BSA, Merck/Sigma-Aldrich, Germany) and 10 ng ml^−1^ TGF-β3 (Miltenyi Biotec, Germany). In order to evaluate the capability of embedded cells inside the dense algMC network to react to the provided chondrogenic factors, their behaviour was compared to algMC-embedded cells cultivated under expansion conditions (ctrl) in DMEM with 1.0 g l^−1^ D-glucose, 9% FCS and P/S. Medium was replaced twice a week.

### Viability and cell number of hCh embedded in algMC

Viability was assessed via live/dead staining (Invitrogen™ LIVE/DEAD™ Viability/Cytotoxicity Kit (ThermoFisher, USA)) and confocal laser scanning imaging (calcein: 488 nm excitation//498–525 nm emission; eh-1: 543 nm//620–700 nm) of z-stacks of a total thickness of 80–120 µm. Confocal laser scanning microscope (cLSM) Leica SP5 equipped with a Leica DFC 500 digital camera and different utilized Apo objective lenses (CS 10x NA = 0.4 dry; CS 20x NA = 0.7 dry; lambda blue 40x NA = 1.25 oil; Leica, Germany) was applied. Quantification of the ratio of viable (marked via calcein staining, 0.6 µl ml^−1^) and dead cells (marked via 1.2 µl ml^−1^ ethidium homodimer 1) was calculated after particle analysis using ImageJ 1.52 p after maximum intensity projection of detected z-stack slices. In biphasic interwoven scaffolds, cell viability was compared between the algMC-CPC interface (gel-CPC) and an algMC intersection (gel-gel) after 1, 7 and 21 days of cultivation. For overview images of entire scaffolds with low magnification, a Keyence BZ 9000 fluorescence microscope was used.

To assess cell number inside the scaffolds, total DNA was quantified and cell number was calculated using a calibration curve of cells collected and frozen at −80 °C prior to scaffold fabrication. Scaffolds were dissolved in 100 mM sodium citrate, cells were lysed via three consecutive freeze-thaw cycles and by sonicating samples in lysis buffer (1% Triton-X100 in PBS) for 30 min on ice. DNA in the lysates was quantified via Quantifluor assay (dsDNA Assay, Promega Corporation, USA) measuring fluorescence signal after reaction at 485/535 nm using a spectrofluorometer (infinite M200pro, Tecan Trading AG, Switzerland). Cell numbers were calculated and graphed as relative to samples (n = 3) collected right after scaffold fabrication at day 0.

Metabolic activity inside 3D scaffolds was proven by observing a color reaction after addition of 500 µg ml^-1^ of 3-(4,5-dimethylthiazol-2-yl)-2,5-diphenyltetrazolium bromide (MTT, Sigma-Aldrich, USA) to cell culture medium and incubation of hCh-laden and cell-free control scaffolds for 4 h; imaging was done via stereo microscope Leica M205C equipped with a Leica DFC295 camera.

### Immunofluorescence staining for cytoskeletal morphology and chondrogenic markers

To observe morphological changes, potential proliferation and chondrogenic marker expression, immunofluorescence staining was applied to hCh embedded in 3D scaffolds. After fixation using 4% formaldehyde in Hank’s balanced salt solution (HBSS, ThermoFisher Scientific, USA), permeabilization in 0.1% Triton-X100, blocking with 1% bovine serum albumin (BSA), different dyes and antibody-mediated labellings were applied: Cell nuclei were stained with 1 µg ml^−1^ DAPI (Gibco life technologies, USA), 1 µl ml^−1^ Phalloidin-iFluor 488 Reagent (Abcam, USA) was used to stain the cytoskeletal F actin filaments. For specific chondrogenic markers, *Anti-aggrecan* antibody (mouse, anti-human, abcam #3778, dilution 1:100) labelled via AlexaFluor 546-tagged goat anti mouse secondary antibody (life technologies A-11003) and *Anti-collagen 2* polyclonal antibody (rabbit, anti-human, ThermoFisher, USA, #PA1-36059, dilution 1:200) detected via AlexaFluor 546-tagged secondary antibody (goat, anti-rabbit, life technologies, USA, #A-11010) were used. Cell morphology and marker expression of hCh was imaged using a cLSM Leica SP5.

### Biochemical analysis of ECM production by embedded hCh

The concentration of secreted sulphated glycosaminoglycans (sGAG) was determined in 1 ml cell culture supernatant collected at day 21 of cultivation after one last total medium change on day 17. Amount of sGAG was evaluated by Alcian blue (Sigma-Aldrich, USA) mediated color reaction after extraction and purification from the supernatants, applying 8 M guanidine-hydrochloride (Thermo Fisher Scientific, USA), SAT buffer (0.056 M H_2_SO_4_ (0.3% v/v); 0.75% (v/v) Triton-X 100, in dd H_2_O), DMSO buffer (40% DMSO, 0.05 M MgCl_2_ in dd H_2_O) and guanidine hydrochloride buffer (4 M Guanidin-HCl, 33% n-Propanol, 0.25% Triton-X 100 in dd H_2_O). Each of these solutions was prepared and applied according to the protocol of a sGAG quantification kit (Kamiya Biomedical Co, USA). For calculation of sample concentration, a standard curve was drawn with a sGAG concentration series of 12.5, 25.0, 50.0 and 100.0 µg ml^−1^ (Kamiya Biomedical Co) after determining absorption of Alcian blue-mediated signal using a Tecan spectrofluorometer (610 nm).

Collagen type II production was determined after scaffold dissolution with 100 mM sodium citrate and cell lysis via three consecutive freeze-thaw-cycles. ELISA using capture antibody mouse anti-chick collagen type II (#7048, Chondrex, Inc. USA), and biotinylated monoclonal detection antibody mouse anti-collagen type II (#7006, Chondrex, Inc.) was applied. Signal based on reaction via streptavidin-HRP (R&D Systems, USA) and 3,3′,5,5′-Tetramethyl-benzidine (Sigma Aldrich, USA) was measured. A standard curve of recombinant human collagen II (Sigma Aldrich, Germany) was set in concentrations between 3 and 200 ng ml^−1^.

### Analysing mRNA expression of chondrogenic/hypertrophic marker genes via qRT-PCR and ΔΔc_T_ analysis

After dissolving the scaffolds in 100 mM sodium citrate at 4 °C, mRNA was extracted from the samples using Qiagen RNeasy Mini Kit (Qiagen, Germany). At least 25 µg of extracted RNA was transcribed to cDNA using SuperScript™ II Reverse Transcriptase Kit (Thermo Fisher Scientific, USA) according to manufacturer’s protocol. The PCR was performed with 1–2 µl cDNA using TaqMan™ Fast Advanced Master Mix for the TaqMan gene expression assay kits COL2, ACN, COMP, COL10, COL1, SOX9 (Table 1); an Applied Biosystems 7500 cycler was used with the following protocol: Initial pre-heating at 50 °C (02:00) (mm:ss) and denaturation at 95 °C 00:20, denaturation at 95 °C for 00:03 and annealing at 60 °C for 00:30 (40 cycles). GAPDH and β-ACT were used as housekeeping genes. Fold change of expression was compared to cells collected at day 0 prior to scaffold fabrication for the gene of interest relative to a housekeeping gene. Comparing mineralized and non-mineralized constructs, relative expression normalised to expression in non-mineralized scaffolds was shown.

**Table 1:** Assays used for gene expression analysis via qRT-PCR (Thermo Fisher Scientific, USA).

Gene	Abbreviation	Assay ID	Amplicon Length
Collagen type I alpha 1	COL1	H200164004_m1	66
Aggrecan	ACN	Hs00153936_m1	91
SRY-box 9	SOX9	Hs01001343_g1	101
Cartilage oligomeric matrix protein	COMP	Hs00164359_m1	101
Collagen type II alpha 1	COL2	Hs00264051_m1	124
Collagen type X alpha 1 chain	COL10	Hs00166657_m1	76
Actin beta	ACTB	Hs01060665_g1	63
Glyceraldehyde-3-Phosphate Dehydrogenase	GAPDH	Hs02786624_g1	157

### Analysis of medium composition during CPC scaffold incubation

CPC scaffolds of 7.0 × 7.0 mm (4 layers), similar to those used in the experiments investigating chondrogenesis in biphasic piled scaffolds, were 3D plotted, treated according to the two-step post-processing regime^21^ and submerged in DMEM with 4.5 g l^−1^ D-glucose and P/S. Four different cultivation schemes were compared by monitoring ionic concentration ranges in the surrounding medium: Two different medium volumes, 1 and 2 ml, were combined with medium changes twice or five times a week. Respective concentrations of collected samples were compared to track the ion concentrations over the observed differentiation period of three weeks. The concentrations of calcium and phosphorus, and therefore ion concentrations of Ca^2+^ and PO_4_^3−^ had been examined using inductively coupled plasma-optical emission spectroscopy (ICP-OES, Plasma Quant PQ 9000 Elite, Analytik Jena, Germany). Therefore, the respective supernatant was diluted in deionised water which was acidified with HNO_3_ in a final concentration of 2%.

### Statistical analysis

GraphPad Prism 8 was used for depiction of data and for statistical analysis. ANOVA with Bonferroni correction was performed to determine statistical significance considering a confidence interval of *p* < 0.05. For experiments comparing between two experimental groups only, unpaired t-test with Welch correction was chosen accordingly.

### Statement

Isolation of human chondrocytes after informed consent and application for experiments were approved by the ethics committee of Technische Universität Dresden (EK 303082014). All experiments and methods were performed in accordance with the relevant guidelines and regulations.

## Results

### Viability of hCh in 3D plotted monophasic algMC constructs

**Figure 3:**
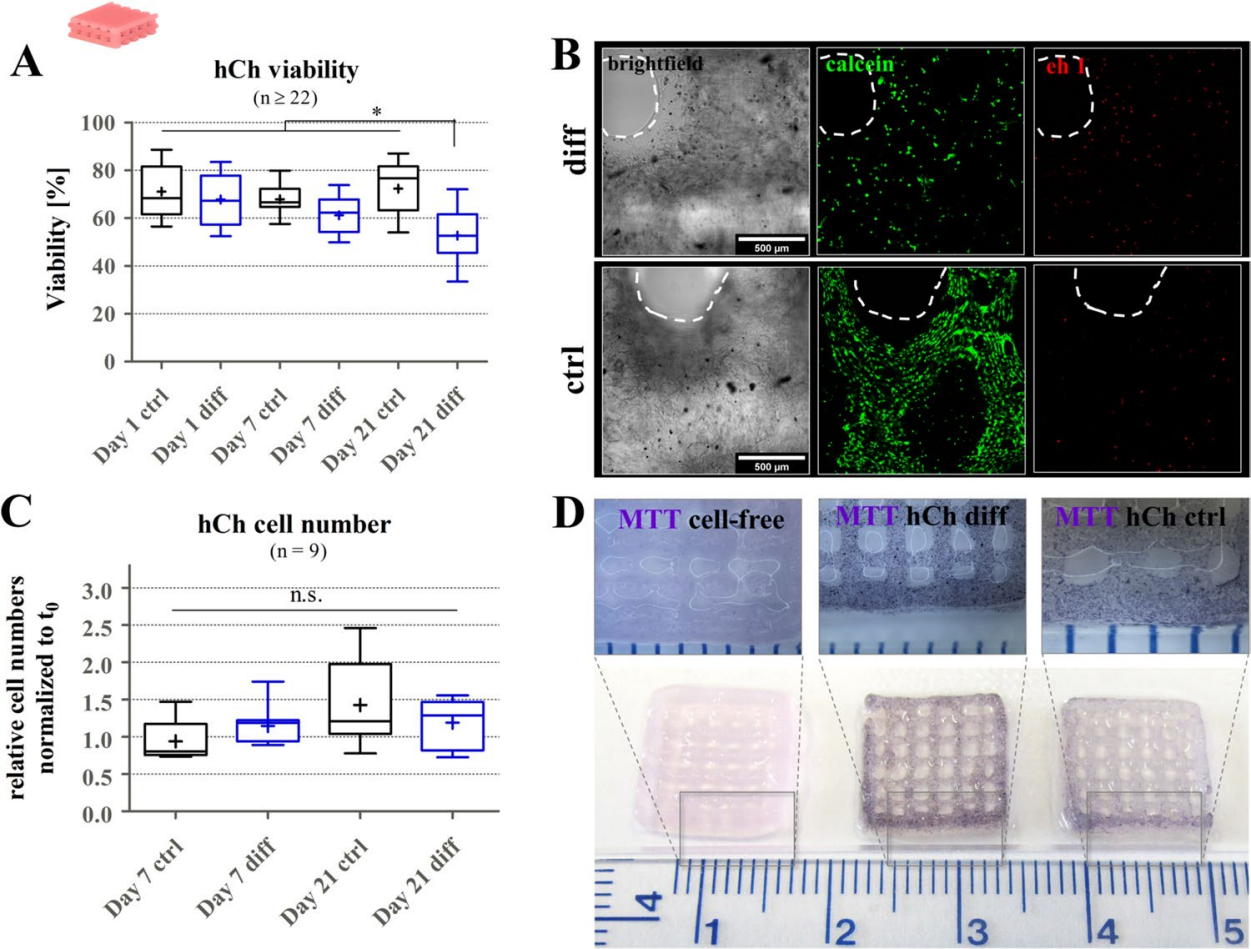
hCh viability in 3D plotted algMC scaffolds. (**A**) Ratio of viable cells and total cell number inside 3D plotted algMC scaffolds, after 24 h (day 1), 7 days and 21 days of cultivation in ctrl and diff conditions, n ≥ 22, *p < 0.05. (**B**) cLSM images of viable cells (calcein, middle column) and dead cells (ethidium homodimer 1, right column) at a strand crossover (brightfield observation, left column) after 21 days of cultivation in ctrl and diff conditions, Scale bar = 500 µm. (**C**) Relative cell numbers, normalized to day 0, after 7 and 21 days of cultivation in ctrl and diff conditions, determined via DNA quantification, n = 9, n.s. = not significant. (**D**) MTT staining of metabolically active cells on day 21 of cultivation in ctrl (right) and diff (middle) conditions, a cell-free scaffold (left) served as control.

To evaluate the applicability of algMC for chondrogenic bioprinting, hCh were mixed into the blend and open-porous 3D constructs were fabricated by 3D plotting and subsequent crosslinking via 100 mM CaCl_2_. Analysis of cell viability revealed rather stable mean viability levels of around 65–75% for both ctrl and diff cultivation conditions (Fig. 3A,B) over one week, while for a late stage differentiation, redifferentiated cells presented a decreased mean viability of 53%. However, cell numbers in all observed conditions remained rather stable comparing different time points to the number of cells initially embedded inside the monophasic scaffolds (6.5–7.0 × 10^5^). No statistically significant difference was found comparing time points and cultivation conditions (Fig. 3C). As a result, metabolically active cells were observed in both diff and ctrl cultivation conditions, visualized via MTT staining on day 21 (Fig. 3D).

### Chondrogenic phenotype in 3D plotted monophasic algMC constructs

**Figure 4:**
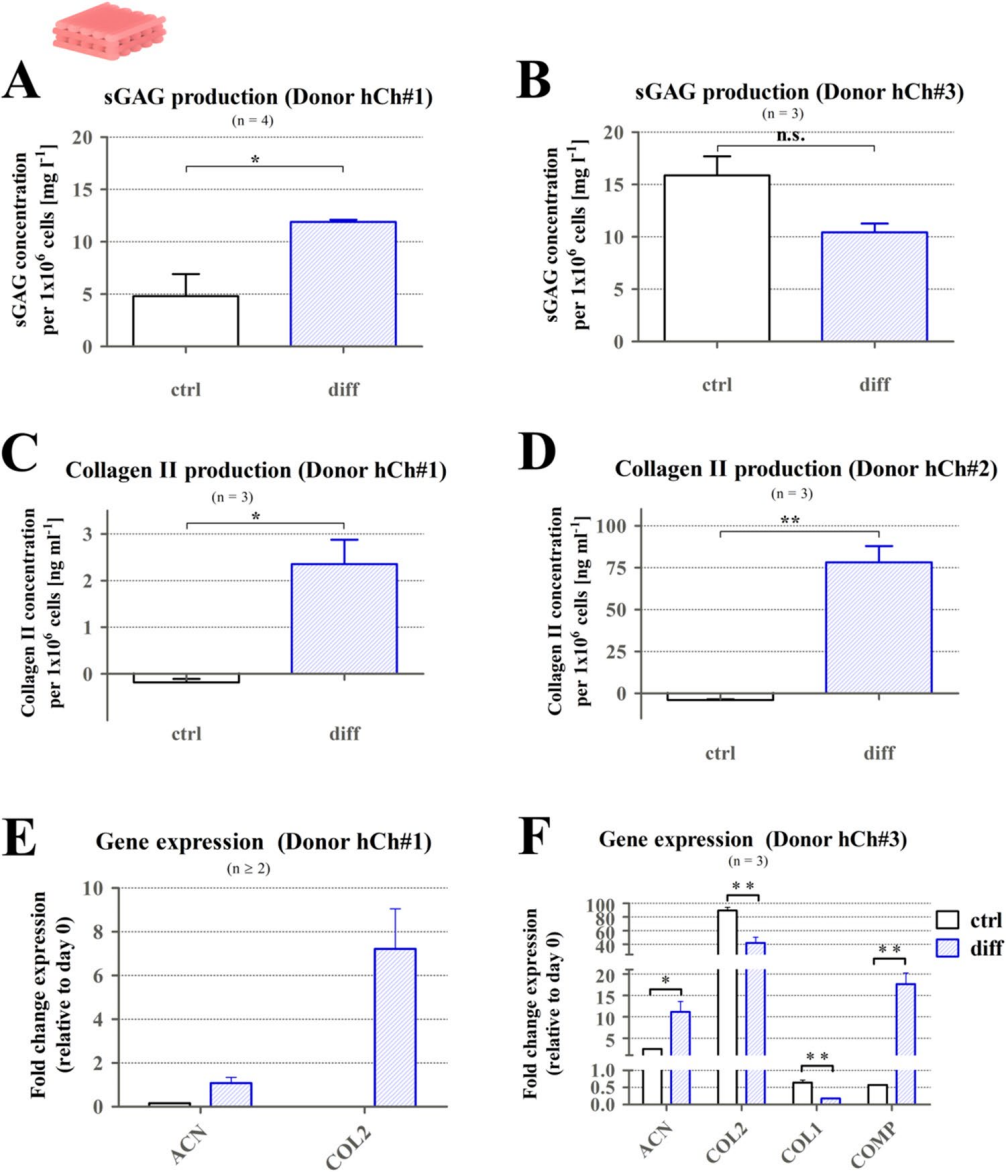
Chondrogenic phenotype inside 3D plotted monophasic algMC scaffolds after 21 days of cultivation. (**A**) sGAG secretion of hCh#1 after 21 days elevated in diff condition (n = 4). (**B**) sGAG secretion of hCh#3 after 21 days revealed similar levels after cultivation in ctrl and diff conditions (n = 3). (**C,D**) Collagen type II production (hCh#1, hCh#2) comparing ctrl and diff cultivation condition on protein level, increased collagen II levels after diff cultivation (n = 3). (**E,F**) mRNA expression for chondrogenic marker genes for donor hChon#1 (E, n ≥ 2) and hCh#3 (F, n = 3), fold change relative to day 0, mean + SEM, *p < 0.05, **p < 0.01.

For required production of their own ECM microenvironment inside the provided 3D architecture, surviving cells were stimulated to redifferentiate to a chondrogenic phenotype over a period of 3 weeks. After 21 days in culture, collagen type II protein production, sGAG secretion into the supernatant, as well as mRNA expression of chondrogenic markers were assessed (Fig. 4). Cells from different donors produced sGAG after cultivation in both ctrl and diff conditions in a concentration of around 10 mg l^−1^ per 1 × 10^6^ cells (Fig. 4A,B), for donor hCh#1 significantly elevated levels in diff condition were detected. Collagen II production was only observed in diff cultivation conditions (Fig. 4C,D).

Comparing the gene expression of relevant markers (ACN, COL2, COL1, COMP) for the two tested donors, chondrogenic marker genes were upregulated in the diff condition compared to ctrl cultivation, except for COL2 expression in donor hCh#3 that was slightly higher in the ctrl condition (Fig. 4C,D). The trend towards chondrogenesis detected for the investigated levels provides the basis for further characterization of hCh fate in combination with CPC inside biphasic structures.

### Morphology of hCh in 3D plotted monophasic algMC constructs

**Figure 5:**
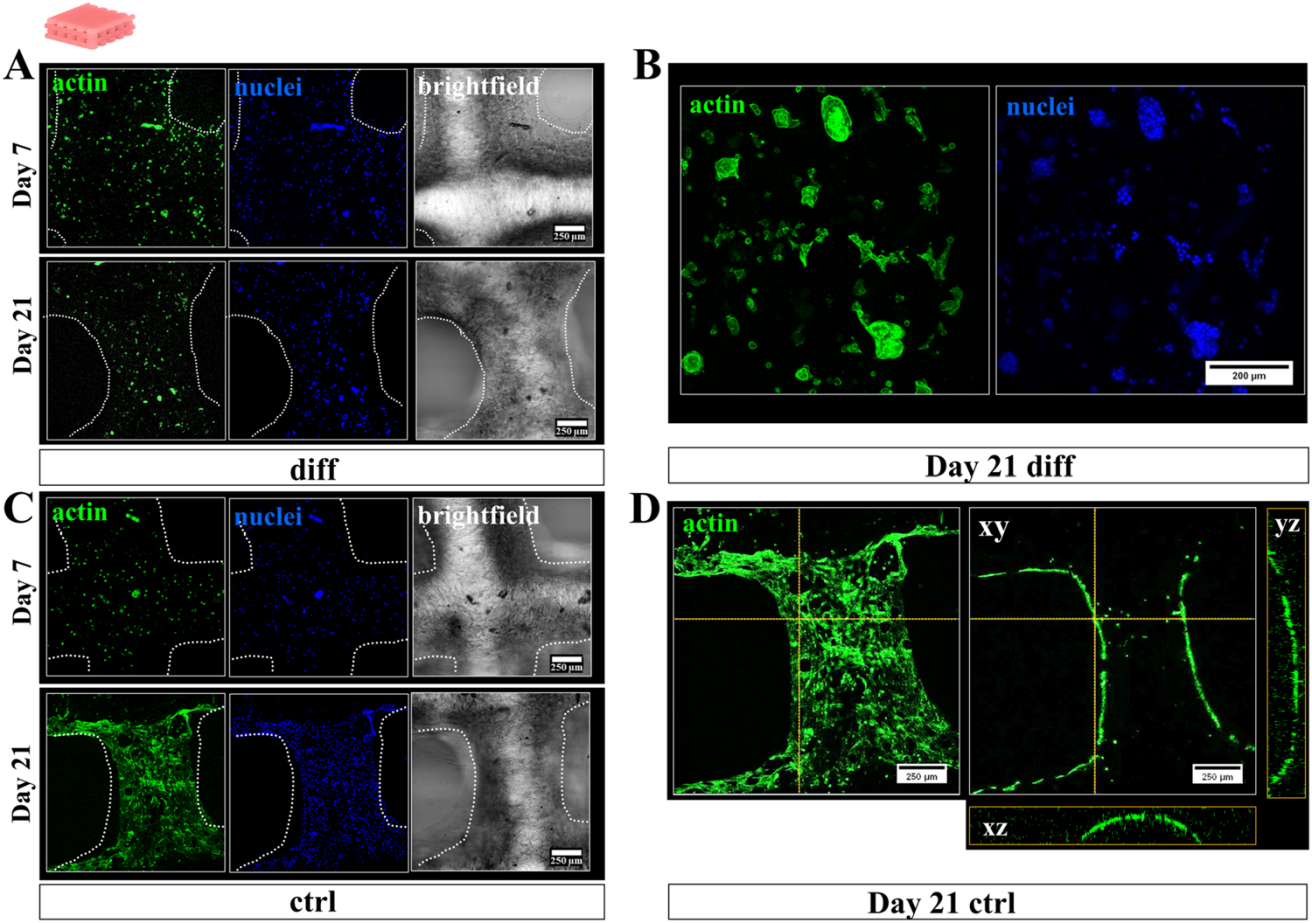
Cytoskeletal morphology and distribution of hCh inside 3D plotted monophasic algMC scaffolds. Fluorescence staining of cell nuclei (blue: DAPI) and cytoskeletal F actin filaments (green: phalloidin) in diff and ctrl condition. (**A,B**) Cells provided with their respective chondrogenic factors remained inside the 3D matrix of the algMC strands after 7 and 21 days, starting to form chondrogenic clusters (**B**) after 3 weeks. (**C,D**) Cells in ctrl cultivation condition started to migrate out of the strand core towards the gel surface colonizing the entire strand. (**D**) Orthogonal views from additionally projected yz and xz perspective on the cell distribution in orthogonal layers of the strand.

Another interesting aspect when comparing morphological appearance of dedifferentiated and redifferentiated hCh was the altered location and morphology of the cells: Cells cultivated in diff conditions remained in a rather round shape and homogeneously distributed inside the 3D matrix while forming clusters of 5–10 cells (Fig. 5A,B). Cells which remained dedifferentiated under expansion conditions (ctrl) started to migrate out of the depths of their algMC matrix to colonize the surface of the deposited strands after 2–3 weeks of cultivation (Fig. 5C,D). Orthogonal views (Fig. 5D) of the strand at one selected position of the cLSM image stack proved the attachment to the strand surface in contrast to the homogeneous distribution throughout the entire thickness of the algMC strand as observed at early time points (Fig. 5A,C, day 7).

### Viability of hCh in 3D plotted biphasic interwoven algMC-CPC constructs

**Figure 6:**
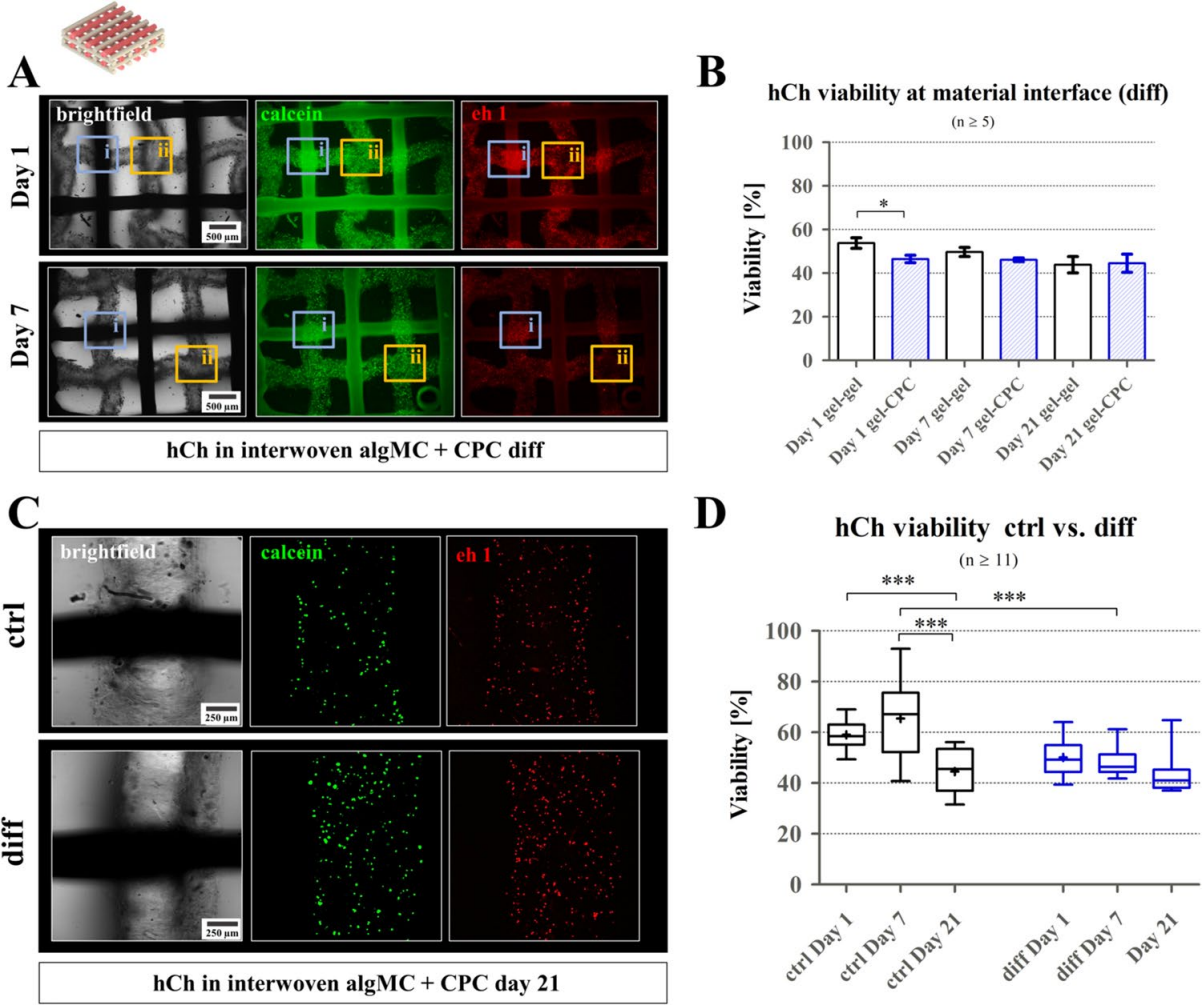
Viability of hCh in 3D plotted biphasic interwoven algMC-CPC constructs. (**A**) Fluorescence images of biphasic scaffolds revealed decreasing cell viability during redifferentiation in close proximity (300 µm) to CPC interface (ii), compared to gel-gel interface (i), scale bar = 500 µm. (**B**) Via live-dead quantification of cLSM images, no statistically significant difference was found comparing differentiating cells at gel-gel interface to cells within a 300 µm distance to CPC strand (gel-CPC). n ≥ 5. *p < 0.05, mean ± SEM. (**C**) Brightfield image of strand intersections, and live-dead stained cLSM fluorescence images of hCh (green: calcein (viable), red: ethidium homodimer 1 (dead)) at strand interfaces of algMC strand and CPC strand inside one scaffold, day 21 for ctrl and diff cultivation, scale bar = 250 µm. (**D**) Quantification of live-dead ratios during 3 weeks of cultivation in ctrl and diff condition, n ≥ 11, ***p < 0.001.

**Figure 7:**
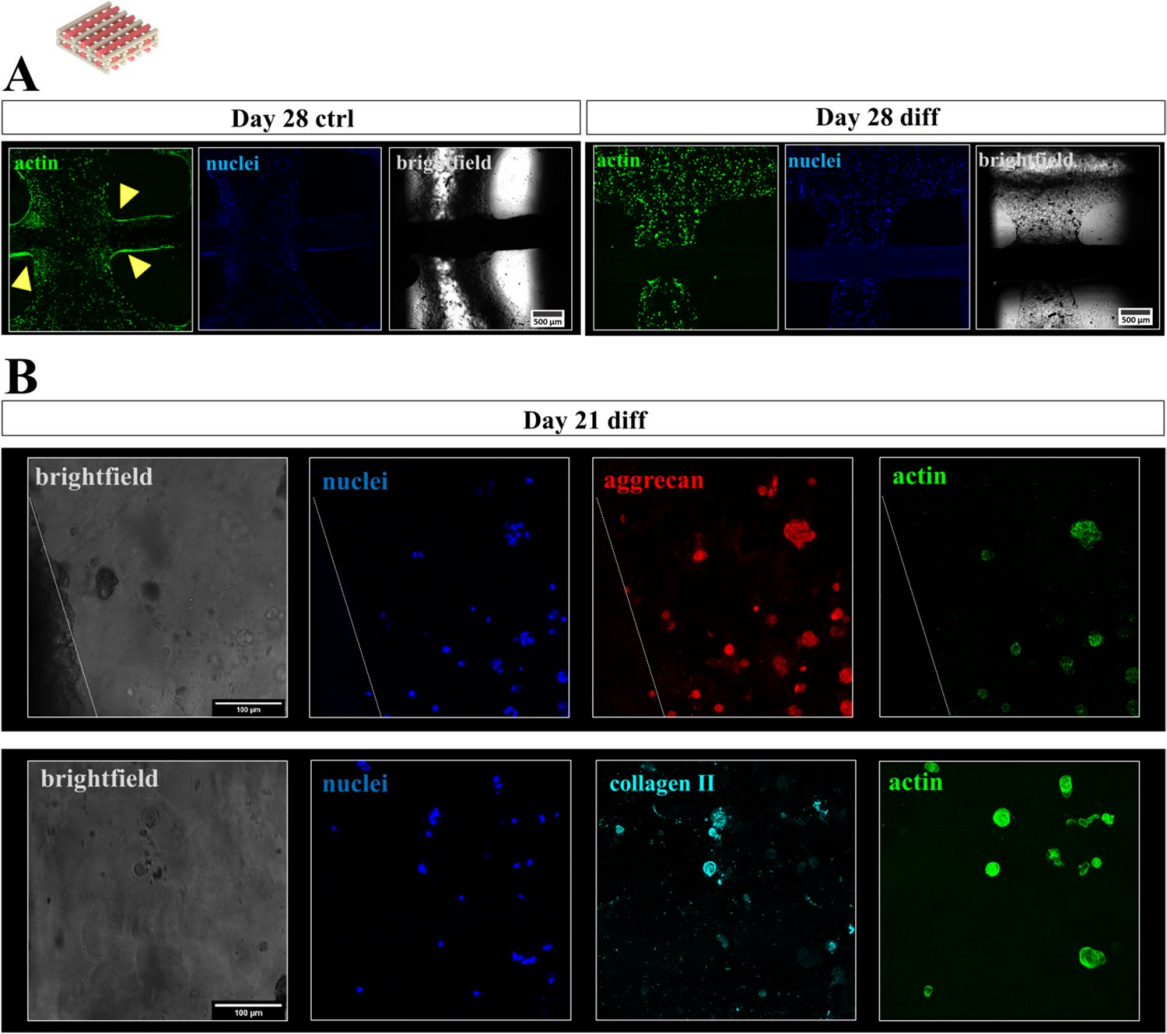
Cytoskeletal morphology, distribution and chondrogenic phenotype of hCh in biphasic interwoven algMC-CPC scaffolds. (**A**) Fluorescence staining of cell nuclei (blue: DAPI) and cytoskeletal F actin filaments (green: phalloidin) at day 28, scale bar = 500 µm. Cells tended to migrate out of the gel and colonize CPC surface (arrows) in ctrl condition (left, day 28) while redifferentiated cells remained inside the algMC matrix phase (right, day 28). (**B**) Fluorescence staining of cell nuclei (blue: DAPI), the chondrogenic markers ACN (top, red) or COL2 (bottom, turquoise) and cytoskeletal F actin filaments (green: phalloidin) in chondrocyte clusters of interwoven algMC-CPC scaffolds in close distance to CPC after 21 days, scale bar = 100 µm.

**Figure 8:**
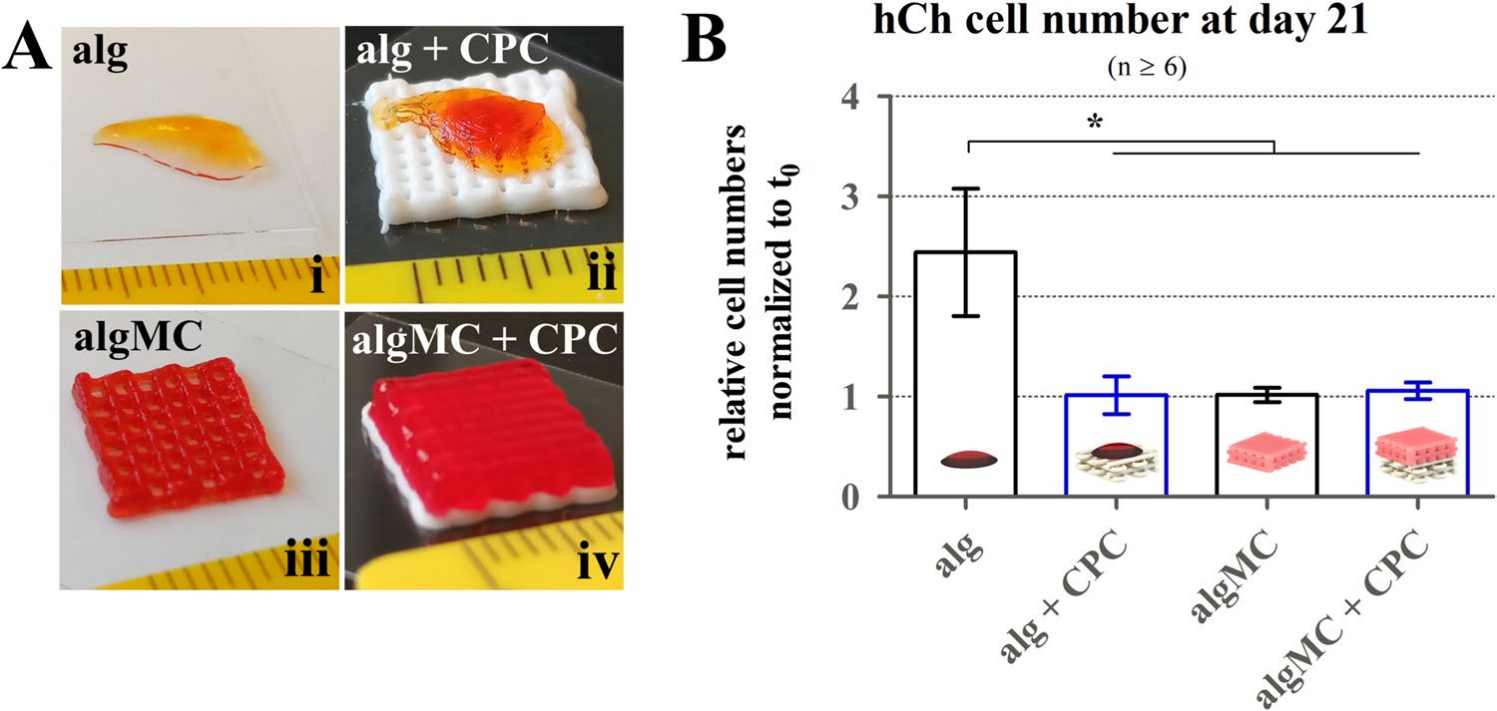
Cell numbers of 3D plotted hCh in algMC in the presence of CPC in a zonal (piled) orientation. (**A**) For co-cultivation of hCh-laden structures in contact to the CPC phase, alg disks (i, ii) and algMC scaffolds (iii, iv) (both visualized via addition of phenol red) were placed on top of plotted CPC scaffolds (ii, iv), scale in mm. (**B**) Relative cell numbers after 21 days of differentiation normalized to day 0 for alg disks and 3D plotted algMC scaffolds with and without contact to CPC (n ≥ 6). mean ± SEM, *p < 0.05.

**Figure 9:**
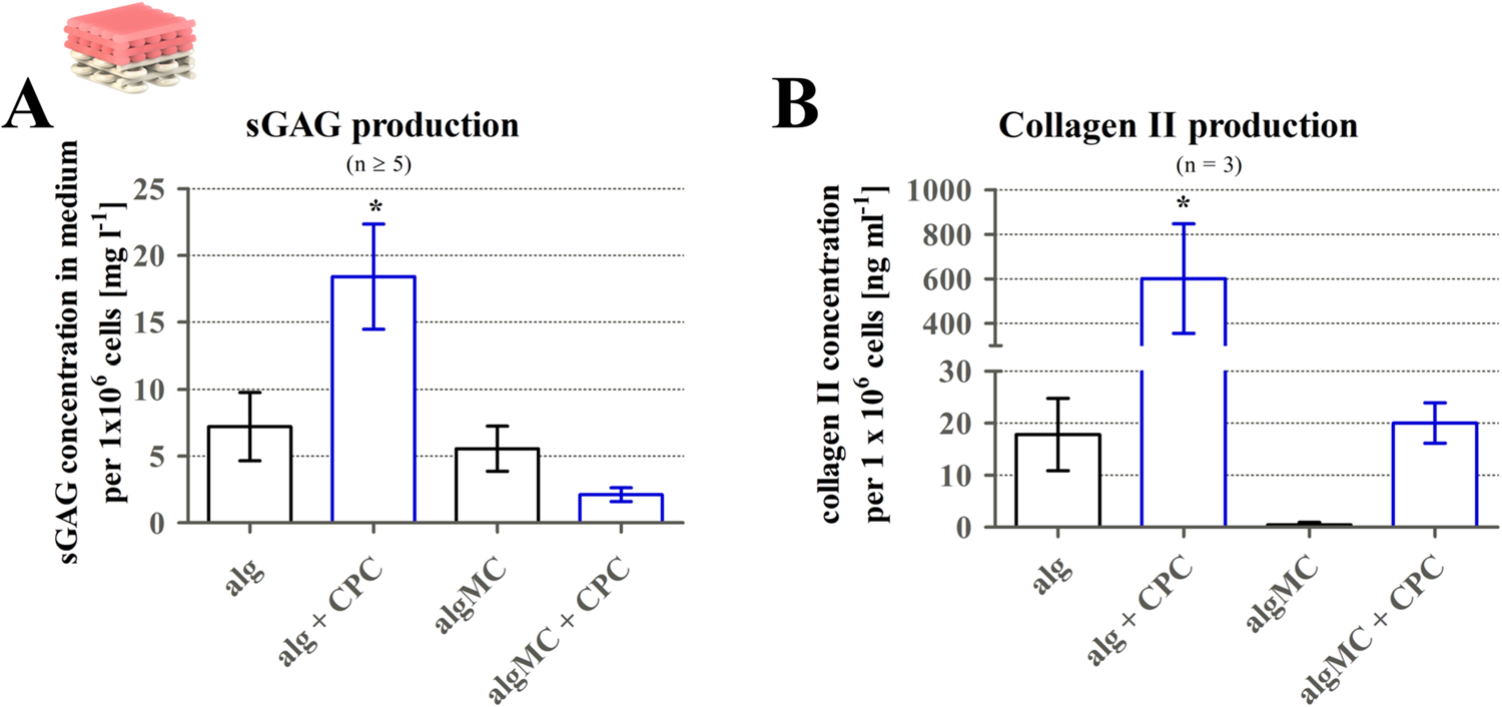
Chondrogenic ECM production in contact to the mineral phase of CPC. sGAG and collagen type II production of hCh inside alg disks and 3D plotted algMC hydrogels after 3 weeks of redifferentiation with or without contact to a CPC phase. Levels of sGAG (A, n ≥ 5) and collagen II (B, n = 3) appeared to be elevated for the alg disk in the presence of CPC. No statistically significant difference was found for algMC scaffolds. mean ± SEM, *p < 0.05.

**Figure 10:**
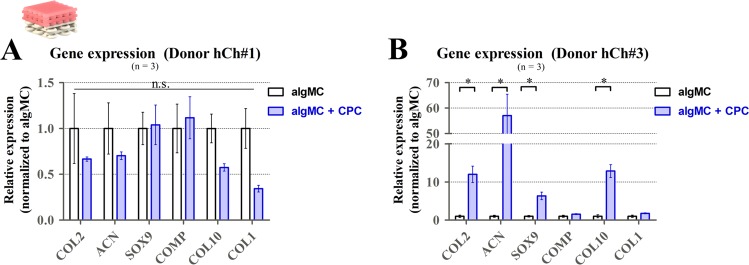
Gene expression of hCh in biphasic piled algMC-CPC scaffolds: Relative gene expression at day 21 of redifferentiation of hCh in algMC with and without contact to a CPC zone. Graphs illustrate relative fold change, normalized to cells incubated in algMC without CPC, mean ± SEM, n = 3, n.s. = not significant, *p < 0.05.

Since we aimed for a full thickness osteochondral model, hCh-laden algMC was combined with CPC in both biphasic interwoven (alternating plotting of hydrogel and CPC strands) and biphasic piled (placing 4 algMC layers on top of 4 CPC layers) pattern. Both these conditions resemble the situation at the osteochondral zonal interface of articular cartilage and calcified tissue. The biphasic interwoven scaffolds revealed the impact of the CPC on the hCh viability and hCh distribution in direct contact to the cell-laden algMC matrix (Figs. 6 and 7) while piled scaffolds were used to investigate the impact of a partly mineralized zone on the chondrogenic phenotype (Figs. 8–10). The latter study included a comparison to an alg disk-based chondrogenesis reference condition, with and without contact to a CPC zone.

In biphasic interwoven algMC + CPC scaffolds (Fig. 6), viability of hCh during differentiation was observed over a cultivation period of 3 weeks in diff medium (Fig. 6A,B) comparing distances higher (gel-gel interfaces, Fig. 6Aii) and lower than 300 µm (gel-CPC interface, Fig. 6Ai) to the next CPC strand. At day 1 after fabrication, number of dead cells was observed as higher at the direct interface of algMC and CPC (Fig. 6A): Quantification revealed a significantly lower viability at gel-CPC interface (46.5 ± 1.3%) compared to gel-gel intersections (53.8 ± 2.5%). Later, cell viability remained constant at around 50% (Fig. 6B), without a reduced cell viability at the gel-CPC interface (Fig. 6C).

Comparing hCh viability in ctrl and diff cultivation conditions, a significantly lower mean viability (64% vs. 48%) was observed for redifferentiated cells at day 7 (Fig. 6C,D). However, cell viability in diff condition remained rather constant while in ctrl condition mean viability dropped to 45% at day 21.

### Morphology, distribution and phenotype of hCh in 3D plotted biphasic interwoven algMC-CPC constructs

Figure 7 presents stainings of hCh-laden algMC strands in direct contact to a CPC strand in a biphasic interwoven scaffold after cultivation in ctrl and diff condition. Similar to the monophasic condition, cells treated with diff medium remained inside the hydrogel matrix over several weeks (shown: day 28); in ctrl medium, the cells tended to migrate out of the hydrogel strand and colonize the stiffer CPC phase (Fig. 7A). After redifferentiation in diff conditions, cells tended to form clusters (3–10 cells) all over the co-fabricated biphasic scaffold expressing the chondrogenic markers aggrecan and collagen type II (shown: day 21, Fig. 7B).

### Chondrogenesis in the presence of a mineral phase in 3D plotted biphasic piled algMC + CPC constructs

To assess the impact of a CPC phase on the chondrogenic differentiation process *in vitro*, biphasic piled algMC-CPC scaffolds were applied (Fig. 8A). The alg and alg + CPC condition (Fig. 8A) were added to this experiment to evaluate the potential effect of CPC on chondrogenesis in an alginate-based standard system. Before investigating their chondrogenic fate, cell numbers were determined: Comparing the relative cell numbers via DNA quantification at day 21 of cultivation in diff condition, the number of cells in plotted algMC gels with and without CPC, as well as in alg disks with CPC, remained stable resulting in a ratio of around 1 comparing cell numbers at day 0 and day 21. In alg disks without CPC, cell number increased by more than a factor of 2 (Fig. 8B).

Figure 9 illustrates the impact of the CPC phase in contact to alg disks and 3D plotted algMC phase on chondrogenic ECM formation (sGAG and collagen type II production) by encapsulated hCh cultivated in diff conditions for 21 days. In the alg disks, sGAG formation per 1 × 10^6^ cells appeared as increased (18.4 ± 3.9 µg ml^−1^) by the presence of CPC but was found lower in 3D plotted algMC gels compared to alg in contact to CPC (Fig. 9A). Collagen type II production (Fig. 9B) was elevated for both encapsulation (alg, algMC) conditions when the constructs were cultivated in contact to CPC.

Gene expression of relevant chondrogenic and hypertrophic markers was analysed for hCh cultivated inside algMC in the presence or absence of CPC (algMC vs. algMC + CPC) under diff conditions for 3 weeks (Fig. 10). In cells isolated from donor hCh#1, expression of all investigated genes was found similar between both groups without statistically significant differences (Fig. 10A). For cells from a second donor (hCh#3), elevated levels of chondrogenic markers COL2, ACN and stemness marker SOX9 as well as for hypertrophic marker COL10 were detected after incubation in contact to CPC; no statistically significant difference between both groups was detected for genes COMP and COL1 (Fig. 10B).

### Calcium and phosphate ion concentrations in the cell culture medium

Due to precipitation processes within the calcium-deficient CPC, calcium and phosphate ion levels in the surrounding cell culture medium are changed, which could also influence the chondrogenic redifferentiation. Therefore, Ca^2+^ and phosphate concentrations were monitored over three weeks for CPC scaffolds incubated in different volumes of cell culture medium (1 ml and 2 ml) with different medium change regimes (5x and 2x per week).

**Figure 11:**
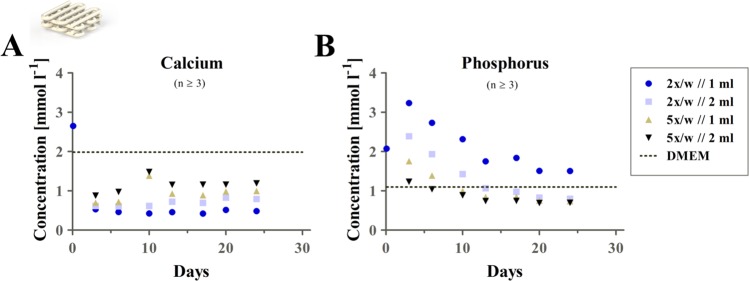
Calcium and phosphate (phosphorus) concentrations. Tracking of the concentrations of calcium (**A**) and phosphorus (**B**) during cultivation of a pure CPC scaffold over 24 days for different medium change regimes. An initially elevated phosphorus concentration was revealed, while calcium concentration was decreased by comparing different cultivation and medium change schemes to the concentration calculated for plain DMEM. First observed sample was measured after 2 h of incubation. The dashed line represents measured concentration in CPC-free, non-incubated cell culture medium, mean ± SEM, n ≥ 3, w = week.

ICP-OES measurement of the samples (shown in comparison to concentrations determined for plain cell culture medium DMEM, Fig. 11 dashed line) revealed a constantly decreased calcium content (Fig. 11A) and an early increase of phosphate concentration (quantified as phosphorus) (Fig. 11B), after an initial increase of both calcium and phosphate after 2 h of re-equilibration after CaCl_2_ incubation as part of the post-crosslinking regime described above. The calculated concentration inside the applied non-treated DMEM cell culture medium was 1.98 mmol l^−1^ for calcium ions, and 1.10 mmol l^−1^ for phosphate ions. Higher medium change frequency and higher volume of medium resulted in reduced effects compared to the standard condition (2×/week//1 ml, blue dot) applied for the cell experiments.

## Discussion

Extrusion-based printing of different materials, different biomaterial inks and bioinks can contribute to the generation of highly specific tissue models and potential patient-individual, tissue-engineered implants. Towards regenerating osteochondral defects by mimicking native tissue structure, a multi-zonal 3D plotted material-cell combination was suggested: The idea was to generate multi-layered tissue substitutes enabling chondrogenesis of embedded cells in different mineralized and non-mineralized cartilage layers. For that, we propose the application of a hCh-laden algMC-based cartilage zone, a calcified cartilage layer combining hCh-laden algMC and CPC, and a subchondral bone layer fabricated solely from CPC (Fig. 1B). The non-cell-laden CPC offers the possibility to seed cells in an additional step after scaffold generation that might be incorporated in future *in vitro* studies towards osteogenic-chondrogenic co-cultivation. Alternatively, *in vivo* the constructs can also be used with non-cell-laden CPC phase since this part of the scaffold is expected to be colonized from underlying bone and bone marrow after implantation due to its osteoconductive properties described above. Cartilage typically hosts encapsulated cells which cannot infiltrate a potential implant making respective tissue engineering concepts necessary.

Due to their additive manufacturing principle, 3D printing technologies can play an important role replacing or improving current clinical treatment of osteochondral defects. Conventional therapy does not offer the possibility to consider geometric aspects or a pre-defined zonal architecture. We expect that the adjustment of the implant dimensions according to a geometry determined via clinical MRI data prior to the surgery can have a major impact on anchorage, integration and performance of an implant. Unlike the commonly used press fit technique applying iliac crest-harvested bone or osteochondral cylinders^36^, 3D printing offers the opportunity to adjust both inner and outer shape of osteochondral grafts according to the patient’s requirements as well as to deposit different materials and cell types in a defined spatial arrangement. Furthermore, the thickness of the individual mineralized and non-mineralized tissue layers can be identified via MRI and considered for the hierarchical, multi-zonal implant which would not be possible otherwise. In addition to these aspects, 3D plotting also enables us to control and define the inner macropore structure. This can be of particular interest for the subchondral bone zone, that consists of different heterogeneous trabecular and spongy cancellous bone zones^37,38^.

Native cartilage does not have direct access to nutrients and oxygen via vasculature. Its metabolism is maintained by diffusion from the synovial fluid supported by mechanical stimulation *in vivo*^5^. Its intrinsic porosity is estimated around 6.0 nm^5^. These aspects represent a significant difference between *in vitro* and *in vivo* conditions as well as between native tissue and artificial matrices: Different studies investigating the *in vitro* performance reported the successful application of open-porous scaffolds^18,39^ or channel-supported alginate hydrogels^12,40^ for chondral regeneration. However, cartilage substitutes for potential *in vivo* application that allows mechanical stimulation, do not necessarily require macroporosity as presented in studies on GelMA-based scaffolds^12,40^. However, macroporosity can support fabrication and therefore stability: In the case of our algMC bioink, it allows for a rapid and homogeneous alginate crosslinking reaction in different layers of volumetric constructs.

Although the thickness of a typical pure articular cartilage surface layer in, for example, a human knee is no more than 3 mm^41^, an entire osteochondral plug with a larger thickness needs to be stabilized accordingly. Applying the algMC bioink, we were able to fabricate 3D constructs with a height of >3 mm. These exhibited great shape fidelity without the need for further stabilization: This was achieved by addition of methylcellulose as a temporary thickener that diffuses from the crosslinked alginate network over time^18,35^. Other studies applied additional polycaprolactone (PCL) strands to stabilize low viscosity hydrogels and to mimic mechanical properties of native cartilage for chondrocyte bioprinting^42–44^. Without external support, these hydrogels did not allow the stable formation of volumetric individual structures, while the algMC blend does. This aspect ensures its strong potency for generating multiphasic volumetric tissue substitutes by 3D bioprinting.

Alternatively, individual and anisotropic printing patterns could be incorporated: These might trigger mechanical properties via porosity and stiffness, resulting also in an altered cell fate^45^, and allowing adjustment of oxygen concentrations in 3D compartments of these constructs. It is known that a low oxygen concentration of 2–5% (physioxia) can be beneficial for chondrogenesis^46^ while in this study a standard O_2_ concentration of 21% was applied. With the underlying results, a physioxia-supported hCh culture in algMC should result in even better ECM production. With the aim of monitoring these oxygen concentrations by real-time measurements, the use of O_2_-sensitive nanosensors integrated into the bioink could be a valuable option^47^.

In the present study, primary human hCh have been incorporated inside the high-viscosity algMC blend for the first time, which had been formerly introduced for the bioprinting of primary hMSC^18^, an hTERT-MSC cell line^21^, bovine chondrocytes^35^ and murine pancreatic islets^48^. Also the suitability of a similar bioink with a lower viscosity based on 2% alginate supplemented with methylcellulose (in alginate:methylcellulose ratios of 1:1 and 1:2) was reported for hMSC bioprinting recently, driving these cells towards chondrogenic differentiation^19^.

This study revealed a viability of hCh after printing of around 70% in monophasic algMC scaffolds (Fig. 3). Comparing this viability to numbers detected for cells encapsulated in alg and harvested cells prior to encapsulation, we illustrated that the encapsulation (blending cells manually into the inks) and/or the extrusion process significantly affects cell survival (Supplementary information, Fig. S1). Our recent study, however, suggests that blending of cells with high-viscosity bioinks might result in a more significant impact compared to the shear stress in the plotting nozzle^49^.

In general, shape fidelity and cell viability over time which were shown around 60% in this study, have to be balanced. Other studies described a lower viscosity of bioinks as sufficient for cartilage formation *in vitro*: These did not allow the fabrication of volumetric constructs without additional support but resulted in a cell viability of around 80%^11^. Markstedt and co-workers presented a cell viability of around 70% at day 1 after 3D plotting of a bioink based on alginate and non-dissolvable nanocellulose^39^. Since the support by the cells’ ECM production is expected as necessary for maintaining a sufficient stiffness inside the hydrogel in addition to the remaining alginate network, a slightly decreased methylcellulose concentration could be an option to improve cell viability with the risk of reducing shape fidelity. Furthermore, more defined, gentle approaches of blending cells and materials should be discussed in the community. In addition, to increase attractiveness of the bioink for cells, specific components of the native ECM could be added.

The cells, from a dedifferentiated state after expansion, redifferentiated within the algMC matrix in the presence of chondrogenic supplements (diff condition) as proven by detected production of ECM components, as well as gene expression analysis of different donors (Fig. 4). These data proved the suitability of algMC as a promising system for chondrocyte bioprinting. In view of our concept for the generation of multi-layered osteochondral tissue substitutes, one major aim of the present study was to evaluate the influence of the CPC mineral phase on the fate of algMC-encapsulated hCh in such biphasic tissue substitutes. Recently, our group reported that the combination of algMC and CPC plotting with viable cells embedded in the algMC bioink, is possible^21^. Herein, for the first time, the biological performance of this promising material combination towards generation of a tissue substitute for the osteochondral interface with an intrinsic biological functionality was characterized.

Our experiments revealed a cell viability of around 50–60% in biphasic interwoven scaffolds (Fig. 6), with a slight drop in close proximity (<300 µm) to CPC at day 1 that is caused by local cytotoxic effects occurring during the CPC setting reaction – the potential impact of pH alterations was discussed before^21^. Later, viability remained stable over 3 weeks of cultivation. Preventing the early impact, a stronger buffer system other than PBS could solve that problem as we demonstrated for algMC dissolved in human plasma towards bone regeneration: The 3-fold buffering system of human plasma^50^ was able to avoid a local drop of hMSC viability at the CPC interface in biphasic interwoven scaffolds^51^. As the cytotoxic effect occurred only in direct contact to the CPC at the crossing points, another option could be the integration of protective cell-free hydrogel strands during the printing process.

When the hCh-laden scaffolds were cultivated in ctrl vs. diff conditions, cells reacted differently: Embedded hCh remained inside the hydrogel strands under chondrogenic stimulation while they migrated towards the strand surface in de-/undifferentiated state in monophasic constructs (Fig. 5). In biphasic interwoven scaffolds, the cells migrated towards the CPC strands and attached and proliferated on the surface of nanocrystalline HAp, formed during the CPC setting reaction, which is in combination with bound serum proteins an attractive substrate for cells^22,24,52^ (Fig. 7).

Mineralization typically is associated with hypertrophic development of hCh and expression of osteogenic rather than chondrogenic markers^53^. Therefore, we wanted to test whether the mineralized CPC phase in the proposed multiphasic scaffolds negatively influences the chondrogenic redifferentiation. In the performed *in vitro* experiments, this suspected effect was not confirmed comparing the phenotype of hCh embedded in algMC and alg with and without contact to a mineral phase of CPC (Figs. 9 and 10). Chondrogenesis was also observed in gels combined with 3D plotted CPC phase. For some experiments, even a supporting effect on chondrogenesis by the presence of a CPC zone was observed. Therefore, we concluded that when encapsulated inside the algMC matrix the hCh are not in direct contact with the calcium phosphate mineral phase, as they merely interact with the ion concentrations of calcium and phosphate inside the surrounding media soaking and diffusing into the hydrogel. ICP-OES measurements indicate ionic alterations in the medium composition over the chondrogenic redifferentiation process in the presence of CPC: incubation of cell culture medium with a CPC scaffold resulted in decreased calcium levels and increased phosphate levels (Fig. 11). The addition of a CPC phase resulted in an impact on the chondrogenesis: This supportive effect of CPC was detected as more pronounced for the cells inside the non-printed alg, i.e. the extent of this effect depends on the gel matrix the cells are embedded in. This might be due to different ionic diffusion conditions in alg and algMC. Since the main goal, however, was the generation of individual 3D constructs, this option cannot be applied. Therefore, future work will focus on balancing shape fidelity and biological performance. In contrast to the rather stable cell number in the other conditions, cells encapsulated in alg disks seemed to proliferate over the cultivation period which is not expected at late stages of chondrocyte differentiation. While algMC, algMC + CPC and alg + CPC cell numbers did not increase, all conditions revealed indications of a certain level of differentiation/chondrogenesis.

Decreased calcium concentrations, as we observed over 3 weeks of cultivation in the presence of the CPC phase, were shown before to boost chondrogenesis and ECM formation^54,55^. The impact of phosphate concentration on chondrogenic differentiation was analyzed in different studies: It was shown earlier, that β-glycerophosphate concentration of 20 mM induced a hypertrophic development of MSC during chondrogenic differentiation^56^. Later it was confirmed that 4 mM extracellular phosphate stimulated the expression of hypertrophic markers (ALP, MMP-3, osteocalcin) and mineralization of growth plate chondrocytes^57^. Wu and co-workers reported that a moderate level of inorganic phosphate supported early chondrogenesis while high levels of phosphate resulted in reduced chondrogenesis and provoked hypertrophy^58^. In our study, the phosphate concentration in the presence of CPC was slightly elevated during the first week of cultivation; at later time points it was found in the range of standard medium composition (1 mM) which might not inhibit chondrogenic differentiation as a low phosphate concentration provided via ascorbic acid (2)-phosphate is typically part of the chondrogenic differentiation cocktail to trigger a positive effect on chondrogenesis of MSC^59^.

However, the underlying results (Fig. 11) suggest that the concentration of calcium and phosphate in the medium strongly depend on the ratio of CPC and medium volume, as well as on the medium change regime. By altering the concentration gradient via the volume of the CPC phase or the medium change conditions, levels of both mineralization and chondrogenesis in the adjacent cell-laden hydrogel zone can be triggered. Therefore, we established a rather simple and versatile way of varying the co-cultivation scheme in contact to CPC.

In this study, we proved that the cells were able to undergo chondrogenesis in mineral-free and mineralized environments and produce ECM components in the selected scaffold designs *in vitro*. The production of their own ECM is required to closer resemble the native tissue *in vitro*, which possesses rather stiff but elastic mechanical properties: Whereas cell-free algMC scaffolds, as characterized before, possess a Young’s modulus of around 35 kPa^21^, native human cartilage has a Young’s modulus of up to 2 MPa^60^. Compressive modulus of a biphasic algMC + CPC scaffold had been determined around 3.0 × 10^4^ kPa before^21^. In this study, we observed the production of the ECM component collagen type II as the main element of the cartilage matrix^5^ under diff conditions and sGAG molecules under both ctrl and diff conditions in similar concentrations, for monophasic constructs, and a sufficient production of ECM components in biphasic cartilage constructs. For both monophasic and biphasic *in vitro* experiments, a high donor variability needs to be reported regarding quantification of gene expression and ECM production. This typical observation could be expected due to the non-controllable specimen source for human femoral head material of mainly osteoarthritic patients of different disease state. The conclusions are scientifically valid and were drawn without overestimating the experimental outcome. Potential further *in vivo* experiments will be necessary to evaluate the clinical potency of the system.

As soon as a potential co-cultivation of cells driven towards both chondrogenic and osteogenic phase comes into play, a spatially defined distribution of materials and local stimulation by spatially defined delivery of differentiation factors in a sustained manner and in sufficient active concentrations will be desired. With respect to this challenge, other studies suggested the incorporation of BMP-2 and TGFβ-3 in different zones^61^. Ahlfeld *et al*. earlier reported about the combination of an alg-based hydrogel 3D plotted in combination with the calcium phosphate cement incorporating specific growth factors for stimulation of angiogenesis^29^. Both materials algMC and CPC^24,62^ allow for the incorporation of specific molecules and biochemical cues. Other options could include the use of coaxial bioprinting^63^ to incorporate differentiation factors in a cell-free core material as a central bioactive depot, with highly adjustable release properties, supporting cells printed in the shell compartment, or the bioink supplementation with ECM components such as specific types of collagen fibers or incorporated sGAG molecules towards tissue-specifically tailored bioinks. These specifications will also allow improving the chondrogenic performance of the applied algMC bioink.

Towards clinical applications, another challenge will be finding suitable ways of fixation of the hydrogel part at the surrounding healthy tissue to keep the implant in the defect site until full integration^64^. Since the attachment of the different materials and layers is quite strong^21^, the conventional press-fit procedure which would be mainly mediated by the subchondral CPC zone, can be applied to keep the implant in place. However, the CPC is a stiff, but also brittle material which might result in a breakdown of the adjusted internal pore structure. Further strategies could include the combination of CPC with reinforcing fibers^65^, the application of fibrin glue or the combination of the printable CPC with other calcium phosphate formulations commonly applied in the clinics. Therefore, the internal architecture will have to be adjusted after identifying the affected regions via MRI, so that a required increase or decrease of the size of the initially measured defect/affected region can be considered in advance.

## Conclusion

In this study, we have presented a novel 3D bioprinting-based fabrication method for generating biologically active individualized osteochondral tissue models. Furthermore, we provided valuable insights into the interaction of mineralization and chondrogenesis in artificial zonal tissue models. The study combines the advantages of multichannel extrusion-based 3D printing with the embedding of autologous cells in hydrogels to form a bioactive, final tissue-engineered substitute with both pre-defined internal architecture and outer geometry. With the presented concept based on CPC and algMC, a stable zonal architecture can be implemented. We could show that encapsulated cells survive and maintain their capability to redifferentiate within algMC, in the absence and presence of a mineral CPC phase. With the findings and explanations about how mineralization and chondrogenesis interact with each other, obtained from the material combination of algMC bioink and CPC, new possibilities for osteochondral bioprinting are opened up. Some aspects about efficient seeding of CPC zone, spatially defined supply with respective differentiation factors and intrinsic improvement of bioink with degradable components such as ECM and sGAG molecules, will be drawn to the focus in future. Therefore, with further progress expected, this will be an essential and successful step towards personalized and biologically active orthopedic implants and mineralized tissue models.

## Supplementary information

Supplementary info.

## Data Availability

The datasets generated during the current study are available from the corresponding author on reasonable request.
