# Supplementary material for: 3D Bioprinting of osteochondral tissue substitutes – in vitro-chondrogenesis in multi-layered mineralized constructs
Source: Sci Rep. 2020 May 19;10:8277. doi: 10.1038/s41598-020-65050-9 (PMC7237416; doi:10.1038/s41598-020-65050-9)
Supplement: Supplementary file 1 — Supplementary info. [file 41598_2020_65050_MOESM1_ESM.pdf]

3D Bioprinting of osteochondral tissue substitutes – *in vitro*-chondrogenesis in multi-layered mineralized constructs

David Kilian, Tilman Ahlfeld, Ashwini Rahul Akkineni, Anne Bernhardt, Michael Gelinsky & Anja Lode

Centre for Translational Bone, Joint and Soft Tissue Research, Faculty of Medicine and University Hospital *Carl Gustav Carus*, TU Dresden, Germany

SUPPLEMENTARY INFORMATION

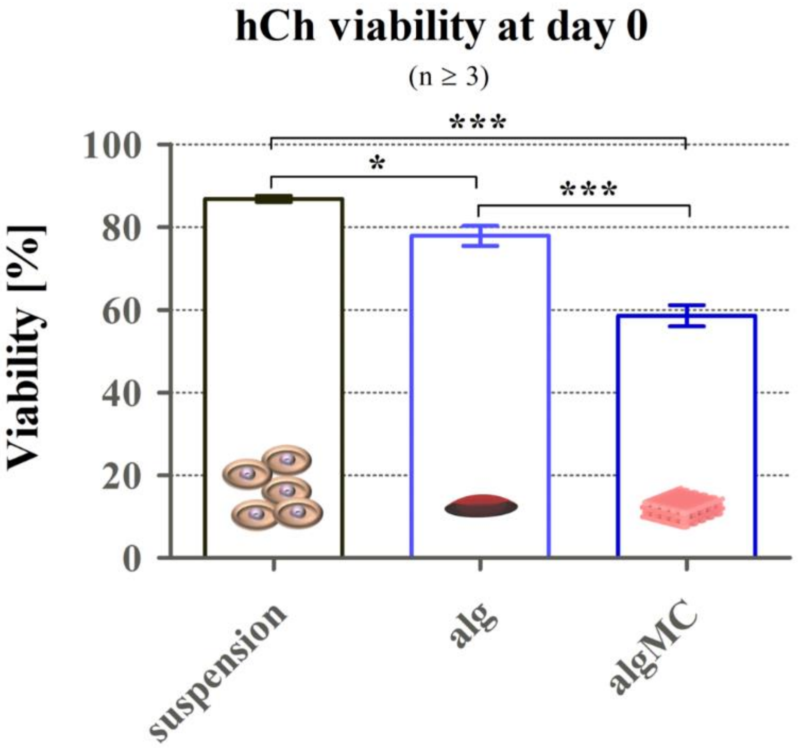

Supplementary figure S1: hCh viability at day 0 prior to and after disk/scaffold encapsulation and fabrication, To evaluate the influence of bioink processing, viability of hCh in the initial cell suspension was compared to viability immediately after embedding into the alg hydrogel and crosslinking as well as to viability immediately after algMC scaffold plotting. This experiment revealed the impact of embedding into alg and algMC, the blending procedure of cells and algMC, the crosslinking via 100 mM CaCl2 and the fabrication process. Mean hCh viability decreased from 86% in the harvested cell suspension to 58% in algMC scaffolds, while in the alg hydrogel 80% of cells survived. mean ± SEM, n ≥ 3, * $p < 0.05$, *** $p < 0.001$.
